# Opinion: Care work, migrant peasant families and discourse of filial piety in China

**DOI:** 10.3389/fpsyg.2023.1280079

**Published:** 2023-12-15

**Authors:** Yan Qin

**Affiliations:** Faculty of International Studies, Southwestern University of Finance and Economics, Chengdu, China

**Keywords:** filial piety, care work, discourse analyses, Foucauldian discourse, Chinese migrant workers

## Introduction

Filial piety, the core pillar of Confucian ethics, is generally referred to as adult children's attitudes and obligations toward their parents (Bedford and Yeh, 2019). In China, like in most Asian countries, adult children are expected to provide support or care to their parents, especially when the parent is diagnosed of fatal diseases. This tradition was born out of the deep-rooted awareness of filial piety from Confucianism, and is sustained by the lack of an all-inclusive social old-care system in modern times. However, in recent decades, the age-old practice of filial piety has been challenged by the massive migration of the labor force from the countryside to the cities due to modernization and urbanization in recent decades. Studies suggest that Chinese migrant families may renegotiate or reinterpret the Confucian virtues of filial piety in order to adapt to the challenges posed by migration and family separation (Choi and Peng, 2016; Chiu and Ho, 2020). Although there have been extensive discussions of filial piety and care burdens of adult children in China, there is a lack of in-depth qualitative research on the updated perceptions and lived experiences of filial care among migrant peasant workers for their end-of-life parents. Longtao He's book, which grew out of his PhD thesis, is a timely work that addresses this gap. By localizing the Foucauldian discourse analysis framework in the Chinese context, He investigated the experiences and perceptions of filial care among migrant peasant workers who came back home from cities to provide care for elderly parents with advanced cancer. This book would be of interest to students and researchers in care work, migrant workers, discourse analysis and filial piety.

### Care work, migrant peasant families and discourse of filial piety in china

The book includes 10 chapters, which falls into two integral parts. The first four chapters are the social, historical and theoretical foundations for this study, while chapters five to 10 are about the specific design and findings of the study.

The book begins with the introduction of the concept of care burden, one of the two key concepts of the study. Here, He introduces the form of informal care and the situation of care burden of Chinese migrant peasant workers and discusses them in the socio-historical context of modern China. However, due to insufficient welfare system provisions for rural families, the decline in the size of family care and governmental response, the care burden for informal caregivers has been exacerbated. Informal caregivers, especially migrant peasant workers, may encounter more difficulties in sustaining care for their elder parents. This highlights the urgency and value of research into the care burdens of rural adult children in taking care of their ill parents.

Filial piety, another core concept of the book, is introduced in the second chapter. Termed by Confucius over 2,000 years ago, filial piety, as a philosophical, theoretical, and ethical concept, “has been constantly shaped and (re)constructed by various social and political forces over time” (p. 43). This chapter looks back at Chinese history and presents a diachronic trajectory of the evolvement of filial piety in China as a philosophical discourse. From the feudal dynasties to the birth of the People's Republic of China, and later to the period after the Economic Reform (1978), it shows the complexity of filial piety, the changes in its meaning and functions imposed by different social-historical backgrounds, and its overall decline over time. Despite its decline, the country is trying to revitalize the concept and value of filial piety among the people to mediate the old care crisis the society is facing.

Chapter Three provides a literature review concerning the two core concepts in the first two chapters, care and filial piety in China. Based on the current literature, the author identifies several research gaps, including the conflation of filial piety and care, the infrequency of migrant peasant workers as research subjects, and the lack of qualitative studies. More importantly, major Western theories on filial piety so far, such as modernization theory and Intergenerational solidarity theory, fail to examine the highly cultural Chinese context of filial piety. This calls for appropriate localized methodologies for research in care and filial piety in China.

Accordingly, in the next chapter, the author proposes and justifies a localized methodology for the research. Drawing from similarities between Chinese philosophies (Confucianism) and Foucault's theories, he identifies a localized Foucauldian discourse analysis tool for this research. This is achieved through examining the differences and more importantly similarities between Chinese and western philosophies, and between Chinese philosophies and Foucault's theories. According to He, Foucault's theories (on power and truth) are similar with Chinese philosophies that “they recognize the complexity of relationality…, incorporate a pragmatic feature… and reiterate the care of the self” (p. 105).

Chapter Five presents the methodological design of the research, including research aims, research questions, data collection methods and procedures for data analysis. This research aims to explore how participants perceive filial responsibility and the way they are influenced by external factors through a social constructionist qualitative approach. The 4-month fieldwork was conducted in the small city of Langzhong in Sichuan Province, China, wherein there has been a marked increase in the incidence of cancer among its citizens. Instrument tools include in-depth interviews, survey questionnaires, observation and field notes.

The findings of the research are presented in the following three chapters as three paralleled themes emerged from the fieldwork. Chapter Six highlights the challenges the caregivers encountered while providing care for their terminally ill parents, including emotional and physical impact, financial impact, work-related impact and unfinished care impact; furthermore, it finds the mediating role of filial piety in participants' care experiences, arguing that, filial piety “could both buffer and exacerbate care burdens” (p. 170) as it serves both as a resource for educating children and the community, and as a hindrance for the care experience since it could provoke stress. Chapter Seven and Eight illustrate the other two themes: the parental sacrifice discourse and the discourse of forgetting (public forgetting and filial forgetting). The participants adopted both two discourses to make sense of their caring roles and construct their filial selves, and filial piety plays a complicated mediating role in the care experiences for parents with cancer.

Based on the findings presented before, the author answers the two research questions of the research in Chapter Nine. Through relating migrant peasant workers' care experiences with the three conceptual similarities between Foucauldian and Chinese philosophies, it is argued that, “The ancient Chinese philosophies may provide a way to incorporate techniques for care of the self and the construction of free ethical subjects that parallel the way Foucault drew on the strength of parrhesia from ancient Greek and Roman genealogical literature” (p. 252).

The final chapter summarizes the key findings and significance of the study and discusses their implications for policy and practice. To fully understand the discourse of filial piety, as He put it, it's important to not simply blame the neoliberal market economic reform for the decline of filial practice and perceptions, and to acknowledge the complexity of filial piety.

## Discussion

This book contributes to scholarship on care work, migrant peasant families and filial piety in several ways. First, the book is a rare combination of care experience, migrant peasant families and advance cancer, each with great socio-economic implications. Compared with urban families, migrant peasant families are marginal and vulnerable groups that are subject to many economic, cultural, and emotional crises. The care work of them for parents with advanced cancer is definitely an understudied topic with great socio-economic and cultural impact. This theme is becoming even more valuable when the current Chinese old-care system is strained and challenged by population aging and dropping fertility rates. As the author argued that, “If migrant peasants' traditionally strongly engrained filial practices and perceptions can change, other demographic groups may also change their relationship to filial piety” (p. 57). The author's explorations into the lived care experiences of migrant workers help create a nuanced picture of the experiences of care workers and their families, highlighting the impact the care work bring and the ways in which care work intersects with culture, history and social norms in modern era. Another strength of the book is its methodological innovations. It has long been a formidable task to examine the discourse of filial piety in China by a single Chinese theory, let alone Western theories. On one hand, endorsed by a long history and changes in feudal dynasties, filial piety carries rich philosophical, cultural and political implications with Chinese characteristics. On the other hand, in the modernization process of China, the country is inevitably influenced by the West in many ways, making the socio-cultural situation more complex. With a solid literature foundation and theoretical justification, this book creates a framework of Foucauldian discourse analysis that is culturally integrated and appropriate for Chinese characteristics, which contributes to the localization of the Foucauldian theories in the Chinese context and to the refinement of Foucauldian discourse analysis as a qualitative methodology.

The book also advances the discipline of gerontology. By examining how migration and changing family structures impact the provision of care for older adults, the work could contribute to the development of culturally sensitive interventions and support systems for aging populations. Furthermore, there is a call for policy changes that address the unique needs of older adults within this population, such as access to healthcare, social support, and financial assistance. As migration and globalization continue to shape societies worldwide, this book might offer comparative analyses or lessons that can be applied in other regions or countries facing similar challenges related to aging, migration, and caregiving.

Despite the contribution in methodology and specific discipline, the author shows reservations about some culturally/politically sensitives issues discussed in the book, such as the pension scheme and the Cultural Revolution. This concern to some extent prevents further exploration and discussion of relevant topics. Meanwhile, a few issues from the book might need more discussion. This book explores the filial practices and perceptions of migrant workers for parents with advanced cancer, while the parent-child relation or parent-child cohesion before the parent's diagnosis, is not mentioned in the study. Filial perception is a complex term that might be affected by various factors, and the parent-children relationship, especially the relationship with parents in the formative years of the children, may play an important role in affecting and shaping the filial perception and practice of the grown-up children. There might be an interesting question to ask that, if the parent is cold and distant from the child throughout childhood, would the adult child still come back home to the countryside to take care of the ill parent? How filial the child will be? Another aspect of concern is the qualitative features of the study. In this qualitative study based on a 4-month fieldwork, more attention has been given to the interview data and questionnaires from the participants. I would recommend more observation and fieldnotes of the scenes and people around to portray a more holistic picture of the external and internal care experience of the caregivers, such as interactions with other patient families of the same ward, comments from the village neighbors, feelings of the ill parents, etc. Last, the discussions concerning the gendered roles of caregivers in migrant worker families, such as the role and duty of “儿媳/er xi/daughter-in-law” in providing care for the ill parent-in-law, could be extended in future studies. Following the topic of “filial daughter and filial sons” by Miller (2004), “the (un)filial daughters-in-law” would be an interesting topic for future research.

Overall, this book sheds light on a meaningful and understudied aspect of Chinese society and carries implications beyond the Chinese context. Against the backdrop of global population aging, filial piety is increasingly taken as a fundamental family and culture value in aging societies (Pan et al., 2022), and the increased importance of filial piety in both cultural discourse and social practice sets the context for filial piety to be politicized (Du, 2021). This study provides an updated, nuanced and complex picture of the filial care experiences of Chinese care workers and provide a solid structure for future research targeting filial care and practices beyond Chinese cultures. I would highly recommend this book to students and researchers interested in care work, migrant workers, discourse analysis and filial piety.

## Author contributions

YQ: Writing—original draft, Writing—review & editing.
